# Views of Australian dental practitioners towards rural recruitment and retention: a descriptive study

**DOI:** 10.1186/s12903-016-0221-0

**Published:** 2016-06-01

**Authors:** Diana Godwin, Ha Hoang, Leonard Crocombe

**Affiliations:** Centre for Rural Health, University of Tasmania, Hobart, Tasmania, Australia; Australian Research Centre for Population Oral Health, School of Dentistry, University of Adelaide, Adelaide, Australia

**Keywords:** Dental practitioners, Oral health, Recruitment, Retention, Rural, Rural health workforce

## Abstract

**Background:**

Despite an increase in the supply of dental practitioners in Australia in recent years, there remains an unequal distribution of dental practitioners with more dental practitioners working in city areas. This is in part due to difficulties in attracting and retaining dental practitioners to rural practice. The aim of this study was to investigate the attitudes of Australian dental practitioners towards what may attract them to rural areas and why they may remain in them.

**Method:**

A descriptive study, utilising telephone, semi-structured interviews with dental practitioners across Australia. Dental practitioners were recruited through their professional associations. Data were analysed using content and thematic analysis.

**Results:**

Fifty participants; 34 dentists, eight oral health therapists, and eight dental prosthetists working in rural and urban areas of Australia. Four main themes were identified: Business Case: concerns related to income and employment security, Differences in Clinical Practices: differences in clinical treatments and professional work, Community: fitting in and belonging in the area in which you live and work, and Individual Factors: local area provision for lifestyle choices and circumstances. The most influential of these themes were business case and individual factors. Smaller rural areas, due to low populations and being unable to provide individuals with their lifestyle needs were considered unappealing for dental practitioners to live. Previous experience of rural areas was highly influential.

**Conclusions:**

The main factors influencing rural recruitment and retention were income sustainability and employment security, and individual factors. Dental practitioners felt that it was harder to earn a sustainable income and provide quality lifestyles for their family in rural areas. Previous experience of rural areas was influential towards long-term rural retention. These factors should be considered in order to develop effective strategies to address the unequal distribution of dental practitioners.

## Background

Australia is one of the most sparsely populated countries in the world. People residing outside capital cities have poorer oral health and less favourable dental visiting patterns than their city counterparts [1]. The differences in visiting patterns may be due to difficulties with access as a result of an unbalanced distribution of dental practitioners between urban and rural areas in Australia [1]. Previous literature has investigated factors which influence rural recruitment and retention of the oral health workforce from Australia and around the world. Working rurally has been linked with desire for a rural lifestyle [2–5], more challenging job opportunities [2, 6], wider range of patients and clinical exposures [2–4, 6], administrative and clinical experience [3, 4, 6], an enjoyable patient base [4], financial incentives [6, 7], personal and professional supportive networks [2, 5, 6, 8, 9], and a sense of belonging to a community [2, 3, 8–10]. However, rural practitioners also experienced a range of negative factors which influenced their decisions to leave rural areas. These included professional and social isolation [2, 4–6, 11–13], limited access to facilities and social activities [2, 4, 13], increased workload and inadequate time off duty [2, 3, 7], limited access to continuing professional development [6, 7], poor access to education services for children [2, 13], limited job opportunities for their partner [2, 3, 8, 11], their own or their family’s dissatisfaction with rural lifestyle and failure to integrate into the rural community [2–5, 7, 8, 14]. Previous studies have found that the most commonly identified indicator of rural practice was prior rural exposure [3, 9, 15].

There have been strategies put in place aimed towards increasing recruitment of both private and public dental practitioners into the rural health workforce. They have included the use of foreign-trained dentists [14], student loan repayment schemes [5, 8, 10, 14] and financial incentives [2, 3, 11]. Strategies aimed at increasing retention of rural dental practitioners included increasing the number of dental students at universities with rural upbringings [11, 16], rural clinical placement programs during undergraduate training [4, 13, 16], and locating dental schools in rural areas [10, 16].

Despite many previous studies focusing on rural recruitment and retention of dental practitioners, a systematic review suggested that more comprehensive research could better investigate the issue by including all types of dental practitioners and excluding other health disciplines such as medicine [15]. Moreover, the literature mostly focused on the views and experiences of rural dental practitioners. As one of the strategies is to encourage non-rural dental practitioners to move to and stay in rural areas, their views should also be explored. The aim of this study was to describe the opinions of Australian dental practitioners towards living and working in rural areas as a part of a further exploratory design research project.

In this study, the term dental practitioner follows the *Australian Dental Board’s* general registration categories of dentists, dental prosthetists, and dental therapists, dental hygienists, and oral health therapists [17]. Therapist, hygienist and oral health therapist participants were combined into one OHT group due to their similarities in provided services. The term urban refers to the Australian Standard Geographical Classification (ASGC) categories [18], major city and inner regional, and the term rural refers to outer regional, remote, and very remote. This study included dental practitioners operating in the private sector, and those working for government clinics in the public sector.

## Methods

This is a descriptive study [19] utilising semi-structured interviews. Ethics approval was obtained from the Tasmania Social Sciences Human Research Ethics Committee (H0013194). Purposive sampling was used to ensure that the sample was representative of the mentioned categories of Australian of dental practitioners, across urban and rural areas, male and female, different age groups and across different states.

Invitation letters and information about the study were sent to the presidents of the four dental associations (Australian Dental Association, Dental Hygienist Association of Australia, Australian Dental and Oral Health Therapist Association, and Australian Dental Prosthetists’ Association) to ask for their support for the study. These four associations agreed to participate in the study. With their approval and support, advertisements to recruit participants were placed in the organisations’ websites and newsletters. Participants were asked to contact the researchers via email or phone if they were interested in participating in the study, they were then asked to use a snowball sampling technique to recruit others [20]. Phone interviews were used because dental practitioners are busy clinicians and were in various locations across the nation. Written consent forms were emailed to each participant. The forms were emailed, faxed or posted back to the researcher prior to the interview.

The interview guide was developed using findings from our systematic review [15] and discussion among the research team to investigate knowledge gaps in the existing literature. It was then piloted with five dental practitioners to make sure that the questions in the guide were appropriate and easy to understand. The interviews were divided into three parts: (i) participant background and training information, (ii) participant views/experiences of why they would or would not practice in rural areas, (iii) participant views on strategies to recruit and retain rural dental practitioners.

The interviews were developed to act as a hypotheses generating tool describing the opinions of dental professionals towards rural practice. All interviews were audio recorded and transcribed verbatim. Each of the interviews were listened back to alongside reading of their full transcriptions for quality assurance purposes. All data were anonymised prior to analysis. Participants are identified only by their professional category, gender, and age. The data were then imported into QSR-NVivo V.10.0 software [21] which assists researchers to store, code, classify and sort qualitative data. Two authors (DG and HH) analysed the data using content and thematic analysis [22]. DG and HH conducted the analysis independently, which involved coding the transcripts, categorising the codes and the generation of themes.

The research team met regularly during data collection and analysis to discuss the process of coding and theme assignment and any disagreements were solved by discussion. The study reached thematic saturation when the researchers identified the content of new interviews repeated that of previous interviews. The researchers used this, as it is a common method of determining if sufficient data has been collected in qualitative research [23].

## Results

Data were collected from November 2013 to March 2014. The interviews varied between 30 and 60 min. Participant demographic characteristics are presented in Table 1. Fifty registered dental practitioners were recruited: 34 dentists, eight dental prosthetists, and eight registered as therapists, hygienists and/or oral health therapists (OHT). Over half were male (56 %) and over three-quarters (78 %) lived in urban areas. Thirty eight participants (76 %) reported having some experience working in rural locations, while there were twelve participants (24 %) who had never worked in rural areas.

**Table 1:** Characteristics of participants

	Dentist (*N* = 34)	Prosthetist (*N* = 8)	OHT (*N* = 8)	Total (*N* = 50)	Percentage %
Location of practice					
RA1-Major cities				(18/50)	48 %
RA1 male	9	3	0	12	
RA1 female	6	0	0	6	
RA2-Inner regional				(21/50)	42 %
RA2 male	10	1	1	12	
RA2 female	5	1	3	9	
RA3-Outer regional				(3/50)	6 %
RA3 male	0	1	0	1	
RA3 female	1	1	0	2	
RA4-Remote				(1/50)	2 %
RA4 male	0	1	0	1	
RA4 female	0	0	0	0	
RA5-Very remote				(4/50)	8 %
RA5 male	2	0	0	2	
RA5 female	0	0	2	2	
Unemployed				(3/50)	6 %
Unemployed male	0	0	0	0	
Unemployed female	1	0	2	3	
Classification of practice					
Urban	30	5	4	(39/50)	78 %
Rural	3	3	2	(8/50)	16 %
Unemployed	1	0	2	(3/50)	6 %
Prior rural exposure					
Yes	25	6	6	(37/50)	74 %
No	9	2	2	(13/50)	26 %
Age groups					
20–34	10	0	2	(12/50)	24 %
35–44	6	0	2	(8/50)	16 %
45–54	4	4	2	(10/50)	20 %
55–64	10	4	2	(16/50)	32 %
65+	4	0	0	(4/50)	8 %
Birthplace					
Africa	1	0	0	(1/50)	2 %
Asia	5	0	1	(6/50)	12 %
Australia	21	6	5	(32/50)	64 %
Europe	6	0	2	(8/50)	16 %
North America	1	0	0	(1/50)	2 %
New Zealand	0	2	0	(2/50)	4 %

**Fig. 1:**
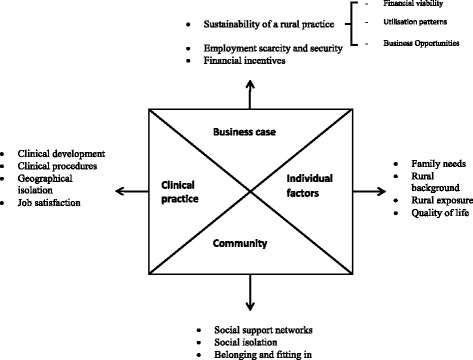
Thematic schema representing dental practitioners’ perspectives on rural recruitment and retention

Four themes emerged from the interviews: business case, clinical practice, community and individual. Summaries of the themes are provided in Fig. 1.

### Business case

When talking about working as a rural practitioner, the majority of the participants expressed their concerns about the long-term sustainability of rural practices, an oversupply of dental practitioners and their views on financial incentives to encourage dental practitioners to work in rural areas.

#### Sustainability of a rural practice

##### Financial viability

Thirty-seven out of 50 participants were concerned about the long-term income security of a rural practice. Due to the high cost of setting up and the daily running costs associated with a private dental practice, participants were concerned about the long-term sustainability and income levels of rural practices. Smaller population size in rural areas was seen as a barrier to rural practice if the local area and the number of patients within it were not large enough to support a dental practice.

*There are more issues than just the money to build a clinic. Um… such as how big is the patient base actually going to be and how sustainable is a dental practice going to be in a particular area. (Dentist, female, 40 yo, urban practitioner-has previous rural experience)*

Household income of rural residents was also seen as an issue for financial viability.

*That’s an issue for rural areas in particular, in that they either don’t have the income to pay for a full time dentist in the area and because it costs so much to set up a dental practice, you need to make sure it’s going to be viable in that area. And that there might not be the population size to afford a rural practice. (Dentist, male, 31 yo, urban practice-has previous rural experience)*

The traditional small business model of the average private practice dentist was also a barrier to rural practice.

*You go up there, and if you were to broke, nobody cares. You’ve gone broke. You’ve lost money, well that’s hard luck for you. (Dentist, male, 66yo, rural practitioner)*

Financial viability of practicing in a rural area also concerned dental specialists and prosthetists:

*I think population, the number, just having the amount of work required to maintain a practice. (Prosthetist, male, 49 yo, urban practitioner-has previous rural experience)*

##### Utilisation patterns

Dental practitioners were also concerned about the utilisation patterns of rural communities. Rural populations were considered to be less likely to seek preventative and routine dental treatment like check-ups, and more likely to seek treatment for problems than urban populations. This pattern of utilisation was considered a barrier to rural practice as it would result in an unviable business opportunity.

*The other thing is that whilst we can talk about shortages and numbers of people, there's still a lot of people who are not choosing to access care so, not all of these small communities can actually realistically sustain a full time practitioner there. (Dentist, female, 52 yo, urban practitioner-no previous rural experience)*

*…And in agricultural areas like this, people compare the price of a crown to the price of an acre of land, and they say well I can make more money out of an acre of land than I can by putting a crown on a tooth [laughs] so they opt for the cheaper options. (Dentist, male, 59 yo, rural practitioner)*

##### Business opportunities

Although some dentists were concerned about the financial viability of rural practice, others saw it as a business opportunity. Some participants decided to work in rural areas because of a perceived need for their services, while others perceived rural practice as lacking the business opportunities available in urban areas.

*I recognised a need in those areas so, um yeah, I’m filling that need, and also a commercial thing. Um, while sometimes it’s not the best commercial decision it does return fairly well, but, fine. (Prosthetist, male, 56 yo, rural practitioner)*

*I wonder whether, you know, it’s financially viable, and for dentists it’s more viable to stay in a central area and have people to come to you. To locate outside a central area, it’s a little bit like a reverse economy. You don’t get as much exposure and you don’t, yeah, you don’t get the same financial return. (Dentist, male, 44 yo, rural practitioner)*

##### Employment scarcity and security

Participants felt that there were too many new dental practitioners graduating from Australian universities. This increase in workforce numbers was thought to be causing employment concerns for dental practitioners in urban and some rural areas, especially for newer graduates.

*… you know that there's going to be an oversupply of graduates, which I think you're going to find its going to be a lot easier to get people to go and do country service, just because, they're going to have to because there's going to be too many unemployed ones in the city. (Dentist, male, 62 yo, urban practitioner-has previous rural experience)*

Many participants (35/50) expressed their concerns with the current job market. There were concerns about limited job opportunities in urban areas and a national oversupply for dentists and oral health therapists. Many felt that as a result of their increasing numbers, the rural recruitment of dental practitioners was no longer a concern.

*I think it is becoming less of an issue with the oversupply of dentists in the metropolitan areas, that people finding, feeling the pressure that they don’t have the options of work in the city. Um, so they're being forced out into country areas anyway. (Dentist, female, 32 yo, urban practitioner-has previous rural experience)*

Some long-term rural dentists mentioned that they used to struggle to find suitable staff for their rural practice, they were currently inundated with eager applications for employment.

*…I, for 30 years used to struggle, I would advertise and I would get absolutely no interest. …[now] I was inundated with applications, you'd only just got to throw, the smallest amount of bait out and there are just kids everywhere just wanting a job. (Dentist, male, 59 yo, rural practitioner)*

##### Financial incentives

Despite believing financial incentives were important in regards to lifestyle maintenance and support, almost half of the participants (23/50) perceived they were not a key driving factor influencing work location decisions.

*Oh, yeah it would certainly, certainly play into it, it would contribute to a positive decision to work in a rural area, but I think there are other intangibles which are, in my particular… which are more important than the financial incentives. (Dentist, male, 35 yo)*

*… they are important, that’s why we go to work that’s why we do what we do, that’s part of the reason why we do what we do. …but that’s also, it’s a trade-off between lifestyle and the financial benefits. I would rather have less of a financial benefit but enjoy the lifestyle that I have. (Prosthetist, female, 49 yo, rural practitioner)*

Some dental practitioners did not see financial incentives as important to encourage dental practitioners to work and stay in rural areas as there could be differences between urban and rural areas.

*I don’t think money brings people to um, you know to areas. It certainly, it doesn’t retain them. I've employed you know various people over the years and paid them exceptionally well, but you know, you see them heading back to the city every second weekend to meet up with their mates and so on, and you know that they're not going to stay. So, you’ve really got to get somebody whose heart is in, in being where they want to be. (Dentist, male, 64 yo, rural practitioner)*

*I think it’s important that you know rural areas that we least have the same earning capacity as metropolitan… I've worked in practices that I've had to go down almost $20 an hour and then in some cases I've moved states where I've had to go down nearly $30 an hour. OHT, female, 49 yo, urban practitioner-has previous rural experience)*

### Differences in clinical practices

When discussing rural recruitment and retention, 43 participants felt that there were differences in clinical practices between urban and rural practice which in turn influenced their decision to move to and stay in a rural area. This included clinical procedures, job satisfaction and professional progression.

#### Clinical development

Dental practitioners did not consider the industry to have important prospects for career progression, advancement and promotion other than that achieved through private practice ownership. Rural practice was considered to be a further limiting factor to career progression and advancement.

*That unfortunately dentistry* via *its nature is a, is a terminal profession. In that there's um not a lot of um opportunities for upward advancement, and that upward advancement is even less so in a rural area. (Dentist, male, 34 yo, rural practitioner, has previous rural experience)*

Rural practitioners who were private owners were often limited in their ability to provide learning opportunities to new clinicians. This was especially problematic when they owned a practice with only one chair, lacking the resources to provide teaching, or the facilities to allow for another practitioner in the practice. Participants felt that new graduate practitioners required professional support and mentoring from senior clinicians in order to increase and grow their skills and confidence levels. This was of particular importance in rural areas, where there is less access to referral pathways and specialist assistance.

*… the graduates don’t tend to have had as much clinical experience as perhaps they once did. And their anxiety to me was “what if I start to do something and don’t know how I finish it?” because you know there's no one there who can help me.” (Specialist dentist, female, 52 yo, urban practitioner-has previous rural experience)*

Providing new clinicians with positive rural exposure and a rewarding working environment was seen to increase their likelihood of long term retention. Dental practitioners who had themselves been in receipt of quality mentoring rated its reciprocation to current new graduates as particularly important. Mentors were able to quickly and effectively increase new graduate’s clinical confidence through rewarding and supportive teaching opportunities.

*I guess the experience that I knew I was going to get when I moved out there [a rural area], my mentor, was really nice and really encouraging. (New graduate dentist, female, 25 yo, rural practitioner)*

Rural practice was considered a barrier to accessing professional support networks. Rural practitioners used the internet to overcome this isolation. Rural practitioners spoke about the increasing professional and personal support which could be available to rural areas through professional associations, using methods such as phone help services, online help, and electronic network communities of rural practitioners.

#### Job satisfaction

##### Professional rewards

Participants [19] mentioned an increased sense of professional satisfaction and pride from their clinical work in rural areas as professional reward. Rural practitioners felt more valued for their services to the local community, that they had status in the local community and enjoyed people ‘knowing who they are’.

*I went out to some really tiny Aboriginal communities for a week at a time, and I just had a ball, I really loved it and you can really tell, like you ask somebody in a small community if you're making a difference and I guess, that played a big part in me choosing to go rural. (New graduate dentist, female, 25 yo, rural practitioner)*

##### Clinical pride

Twelve participants mentioned that being dental practitioners in a rural area brought clinical pride, reinforced by their sense of thanks and adoration received from patients. Rural practitioners felt pride in what they provided to their local communities, which was often enhanced if they were the only dental services available in the area.

*I found it much more fun to practise in those areas, much more rewarding you’d have people with serious um dental conditions which were affecting their medical health rather than just a simple broken tooth. (Dentist, female, 32 yo, urban practitioner-has previous rural experience)*

##### Clinical procedures

Rural practice was considered an avenue for requiring increased clinical skills, as there were less available referral pathways to other health practitioners. Rural practice for younger practitioners was considered a fast way to up skill and learn clinical treatments quickly.

##### Geographical isolation

Close to half [21] practitioners were concerned about geographical isolation from urban centres. This was seen as a barrier for access to professional services due to increased travel time and costs, and increased amount of time taken away from work to attend sessions in urban centres.

*I would definitely make sure that there was an education, peer support network for rural practitioners, um, I'd make sure there was some sort of assistance for their greater out of pocket costs. (Dentist, female, 54 yo, urban practitioner-no previous rural experience)*

Support from professional associations, professional networking and peer group support was thought to be harder to access in rural areas than urban areas due to a lower number of health professionals.

#### Community

Fitting into the local community played an important role in dental practitioners’ decisions to move and stay in a rural area. The participants spoke about who they were, what they valued, and how they provided for and were provided for in their social networks and communities.

##### Social support networks

Social support networks were rated high on the list of important factors which influence participants’ decisions to move to rural areas. Regardless of their upbringings, either urban or rural, participants felt connected and comforted by the presence of family and friends. It was the strength of their connectedness to outside of work social contacts which enabled them to enjoy and value their lifestyles. Family ties were mentioned by 13 participants as shaping where they chose to work.

*Lifestyle, I think, and family. I think just know where you like to live, I'd prefer to live in a rural area. (Prosthetist, male, 52 yo)*

##### Social isolation

All interview participants mentioned the high importance of belonging and fitting in their local community. There were increased problems associated with moving away from support areas. Participants stated that moving away from where they currently live and where they have pre-established social support networks had simply not occurred to them. Others had not ruled out a move, but did not have enough incentive to relocate and were fearful, and concerned about loneliness and community integration. Some rural practitioners who were working away from family and friends described it as a lifestyle choice, one which was overcome by regular travel to urban areas.

*The lifestyle I think I was sort of itching to get back into the CBD [city area]. Um mainly because of you know being closer to friends and family. (Dentist, male, 59 yo, urban practitioner-has previous rural experience)*

Community was mentioned as a negative aspect of rural practice in relation to ethnicity and individuals who were not ‘local’, and as a result felt that they were not accepted by the rural community. However, community engagement was a positive factor for rural practice for dental practitioners, providing social support, networking and social activities.

Some participants believed that rural areas had a stronger sense of community engagement than urban areas. Practitioners felt that having an established practice and being a visible outside of work personality in the local community was key to retaining dental practitioners in rural areas. Some urban dental practitioners who moved to rural areas found an initial sense of isolation and loneliness, but with social and community integration, they assimilated into the community and gained a sense of belonging. This ‘urban’ identified participant explains their journey into the local community.

*I live in a small town now and I don’t think I've ever lived somewhere so social in my whole life. It’s a lot more social because you end up making your own fun. (OHT, female, 58 yo, rural practitioner)*

### Individual factors

Participants were asked about their personal backgrounds. Individual factors such as backgrounds, family needs and quality of life played an important role in dental practitioners’ decisions about working in rural areas.

#### Rural background

Participants who had a rural background were likely to mention the simple enjoyment of living and working in smaller communities. Participants who self-identified as ‘rural’ were likely to have grown up in a rural area or had spent a major proportion of their working lives in rural areas.

*I've got this bigoted view that rural people want to work in rural areas, and people who grew up in the metropolitan areas probably want to work in metropolitan areas. And the reason I think that is, you couldn’t pay me enough to get me to work in the metropolitan area and yet, yet we sort of, you get people going around saying, “so why won't they move to the country?” and I say oh well the same reason I won't move to the city.” (Dentist, male, 63 yo, rural practitioner)*

Participants who self-identified as ‘urban’ felt fearful about rural practice as a result of not having had previous experiences or exposure to rural areas. Urban background practitioners mentioned never having considered rural practice due to already having employment opportunities in their local area.

#### Rural exposure

Twenty-two participants mentioned rural exposure prior to working as a dental practitioner in a rural area. This was most likely to occur during their education through rural clinical placement programs, during previous rural work experiences through contractual work, or their own rural background and their partners’. This allowed practitioners to develop a true sense of the rural lifestyle and the realities of rural practice and community integration. Rural exposure was felt to increase the likelihood of initial rural recruitment and longer term rural retention.

*I think what they're doing at the moment that’s having universities in rural settings. It’s sort of giving the students the opportunity to actually be exposed during that 5 years training. And also that may give them the opportunity to sort of go, look maybe this isn’t that bad after all, it’s actually quite a nice experience personally. (Dentist, female, 33 yo, urban practitioner-has prior rural experience)*

#### Family needs

The actual location of their practice was more often influenced by wider family concerns. The provision of lifestyle rewards for partners and children were the most important factors. Rural practice was considered a barrier to accessing appropriate high school and tertiary education.

*…our son’s education, he was getting to 12 years of age and it was a choice either he went to boarding school or we would relocated. And we looked at the alternatives and boarding school was not one that we welcomed so we relocated. (Dentist, male, 70 yo, current urban practitioner-has extensive previous rural experience)*

Dental practitioners also identified their significant others as having influence over where they chose to work and for how long. Rural areas were considered more difficult for employment opportunities for couples as two professionals. However, having a partner or a spouse with a rural background or upbringing could increase the likelihood of rural practice.

*… The big thing that I would emphasis would be the lack of job opportunities for partners, because partners are likely to be educated and you know professionals and so, it’s certainly a major factor for a lot of people. (Dentist, female, 32 yo, urban practitioner, has previous rural experience)*

#### Quality of life

Forty participants mentioned quality of life when discussing rural recruitment and retention. Participants referred to lifestyle rewards and rural enjoyment as quality of life.

*…it’s mainly just lifestyle rather than work, where I, that determines where I live. (Prosthetist, male, 52 yo, urban practitioner-has previous rural experience)*

Rural practitioners enjoyed what they called ‘rural lifestyle’ this was considered separate from ‘city lifestyle’. This term referred to feelings of a more relaxing and laid back daily life. Lifestyle rewards were considered in conjunction to all types of financial incentives, and were thought to be more important provided the financial incentives allowed a reasonable income. Lifestyle rewards were considered to be of key importance for rural practitioners to facilitate long-term retention.

## Discussion

These findings confirms some factors from previous studies, and it adds that private practitioners were concerned about the future income security when considering to move to a rural practice. This was not a concern for practitioners in the public sector as they were salaried employees. While other factors such as enjoyment of rural lifestyle [2–5], social isolation [2, 4–6, 11–13], limited access to facilities and social activities [2, 4, 13], limited access to education services for children [2, 13], and limited job opportunities for partners [2, 3, 8, 11]; could be negotiated, ignored or ‘solutions’ found, the failure to reach an appropriate income level to support one’s family was not able to be substituted with other factors. Participants expressed concern that some rural areas did not have large enough population numbers to adequately financially support a full-time private practitioner. Australia is one of the most sparsely populated countries in the world. Nearly 90 % live in urban areas (more than 1000 people) [24]. However; in 2011, 1.8 million people lived in rural areas outside any defined towns or localities [24]. Tennant and colleagues [25] proposed that there was a minimum population level for communities which is required in order to support a full-time dental practitioner and that many areas in Australia do not fulfil this population requirement. This is further complicated by differences between urban and rural clinical work, including lower routine visiting patterns [26] and a higher likelihood of emergency treatments [27]. In Australia, dental services are largely provided in the private sector (85 %) [28], and the burden of payment falls to the individual. The cost of treatment is a common reason for people to avoid dental treatment, leaving a large proportion of the community with untreated dental issues [2, 29]. Given the manner in which dental care is provided, a private dental practitioner requires a larger patient base than a medical practitioner does to financially support their practice and many widely-dispersed rural areas in Australia do not have the population size to support a full-time dental practitioner [30].

Strategies such as higher salaries and financial remuneration [2, 3, 11] to encourage rural practice would attract public dental practitioners. A recent program in Australia provides relocation incentives and infrastructure support grants to private dentists who relocate to provide general dental services in regional and remote locations [31]. However, for many participants in the current study, there had to be assurance of long-term financial security from the work location before other factors were considered. This is a complex issue which requires flexible, practical and different models tailored for rural oral health care delivery for individual communities [26], mobile clinics and tele-dental services [32].

Another important finding from this study was that individual factors played an important role in influencing rural retention [3, 33]. These aspects included the successful formation or pre-existence of strong social bonds to the local community and personal enjoyment of rural lifestyle [3]. This was facilitated by the local rural area being able to ‘provide’ certain lifestyle necessities for the individual and their families. The most important of these ‘provisions’ were family concerns: quality schooling opportunities for children [2], and sufficient employment opportunities for partners [2]. Furthermore, having prior rural exposure and positive experiences of rural areas for themselves and their partners influenced later work location decisions [9, 34, 35]. This is known as the Rural Background Effect [36, 37]. The strategies which supported this factor were: increasing the number of dental students at universities with rural upbringings [11, 16, 35], rural placement programs during undergraduate training [4, 13, 16], locating dental schools in rural areas [10, 16, 35]. Retention issues are extremely complex and so too would be the solution, with issues to be addressed in the future being avenues to facilitate employment opportunities for the spouses of relocating dental practitioners, sense of belonging in rural communities and social engagement with local populations.

The limitations of this study were due to the nature of volunteer participants, there was a higher than average proportion of rurally experienced dental practitioners donating their time for the interviews. Using snowball sampling could introduce bias as individuals who know each other could share similar characteristics and opinions. The higher number of dentists compared with OHT’s and prosthetists could mean that factors which were influential for dentists in comparison to other dental practitioners may have been overly addressed. Further rural dental practitioner workforce research with a larger sample size is required to assist policy makers plan for more equitable access to oral health care for rural Australians.

## Conclusions

The main factor influencing rural recruitment and retention was financial sustainability. Dental practitioners felt that it was harder to earn a sustainable income and provide quality lifestyles for their family in some rural areas. Previous experience of rural areas was considered to be highly influential towards long-term rural retention.
